# The “Wireless Sensor Networks for City-Wide Ambient Intelligence (WISE-WAI)” Project

**DOI:** 10.3390/s90604056

**Published:** 2009-05-27

**Authors:** Paolo Casari, Angelo P. Castellani, Angelo Cenedese, Claudio Lora, Michele Rossi, Luca Schenato, Michele Zorzi

**Affiliations:** Department of Information Engineering, University of Padova, via G. Gradenigo 6/B, 35131 Padova, Italy; E-Mails: castellani@dei.unipd.it; herzog@dei.unipd.it; loraclau@dei.unipd.it; rossi@dei.unipd.it; schenato@dei.unipd.it; zorzi@dei.unipd.it

**Keywords:** wireless sensor networks, testbed, ambient intelligence, wireless reprogramming, radio channel model identification, localization, tracking, implementation

## Abstract

This paper gives a detailed technical overview of some of the activities carried out in the context of the “Wireless Sensor networks for city-Wide Ambient Intelligence (WISE-WAI)” project, funded by the Cassa di Risparmio di Padova e Rovigo Foundation, Italy. The main aim of the project is to demonstrate the feasibility of large-scale wireless sensor network deployments, whereby tiny objects integrating one or more environmental sensors (humidity, temperature, light intensity), a microcontroller and a wireless transceiver are deployed over a large area, which in this case involves the buildings of the Department of Information Engineering at the University of Padova. We will describe how the network is organized to provide full-scale automated functions, and which services and applications it is configured to provide. These applications include long-term environmental monitoring, alarm event detection and propagation, single-sensor interrogation, localization and tracking of objects, assisted navigation, as well as fast data dissemination services to be used, e.g., to rapidly re-program all sensors over-the-air. The organization of such a large testbed requires notable efforts in terms of communication protocols and strategies, whose design must pursue scalability, energy efficiency (while sensors are connected through USB cables for logging and debugging purposes, most of them will be battery-operated), as well as the capability to support applications with diverse requirements. These efforts, the description of a subset of the results obtained so far, and of the final objectives to be met are the scope of the present paper.

## 1. Introduction and Project Objectives

Currently, one of the major research efforts in the field of network architecture and communication is devoted to the development of the so called *ad hoc networks*, which are characterized by the ability to communicate and coordinate without the need for a centralized base station to undertake these tasks. This may be required for a number of reasons including, but not limited to, emergency scenarios, infeasibility or impracticality of wired layouts (e.g., at business meetings), battlefield deployments, and so forth. The very fact that nodes are not located and configured based on a priori network shape and architecture, but are instead placed where there is a specific (*ad hoc*) need, gives rise to the name.

*Wireless Sensor Networks* (WSN) are a very specific class of *ad hoc* networks, composed by a number of devices spread throughout a given area, and capable of collecting information about the surrounding environment, of processing this information, and of circulating it through the network via a wireless communication channel. In [1] the main features of WSNs are discussed with reference to the wide range of applications in which they can be employed. In this context, it is useful to highlight how the WSN approach perfectly fits the perspective of ambient intelligence in large, possibly city-wide scenarios. First of all, let us recall that wireless sensors are usually very small nodes with a limited set of capabilites. They usually incorporate by default a set of environmental sensors (temperature, light, humidity), a programmable general-purpose microcontroller, some RAM/ROM units and a radio transceiver unit. Recent implementations of wireless sensors also incorporate a serial connection (e.g., through a USB interface), which is used for programming, but can also be employed for power supply. By means of wireless communications, the sensors can be organized into a network that cooperates in tasks including, but not limited to, environmental monitoring and control, alarm dissemination, localization and tracking of moving objects, and ambient intelligence in general. The latter definition encompasses a number of applications involving complex interactions between a user, the network, and the environment, possibly including some specific actuation devices that might be interfaced to the sensors in order to carry out a certain task. For example, a wireless sensor with a hardcoded identity footprint could communicate with the network so that its movements can be tracked: such a sensor could, e.g., be attached to a piece of luggage, to make sure that it is routed toward the correct destination by airport logistics and that, in case of errors, its route can be quickly reconstructed, in order to allow timely recovery.

In WSNs, the energy efficiency aspect is of paramount importance. In fact, it should be noted that wireless sensors are mostly powered through external batteries or battery packs. As this supply is limited, energy efficient operations and communications must be pursued, so that battery replacements on the sensors are as infrequent as possible, in order to keep power supply and maintenance manpower costs low. For this reason, networking protocol design as well as application design must carefully account for the limited processing and storage capabilities of the microcontroller: therefore, the further delay that would be caused by complex processing tasks should be explicitly considered in light of, e.g., the timing of communication protocols. In the following, we give more details on the typical configurations and peculiar features of WSNs, in order to provide a wider characterization of their uses and behaviors. In general, a WSN is characterized by being:

- partly stationary: the vast majority of the deployed nodes are fixed and constitute the infrastructure of the network. They are strongly *embodied* within the environment, but allow the presence of a fraction of mobile nodes that contribute to the overall information gathering process with local and often volatile knowledge;
- wide: the possibility of having a large number of sensors deployed either randomly or manually on a vast geographic area without issues related to cabling or a priori hierarchical communication structures, allows the introduction of pervasive (but not invasive) intelligence in the environment;
- homogeneous: as opposed to the general ad hoc approach, where the network is made by grouping devices of potentially very different nature, most WSNs are composed by nodes which are similar to one another. For example, in the specific case of an environmental monitoring application, some nodes may be equipped with temperature sensors, other nodes with humidity sensors, but all bear similar communication devices and processing capabilities; it should be noted, however, that heterogeneous networks have been conceived as well, where, e.g., a large number of nodes perform sensing, a few expensive nodes provide data fusion and filtering, and the node differences in terms of computational capabilities and links are exploited for networking purposes [2].

As will be made clearer in the following sections, these three aspects represent canonical issues that are kept in focus during the design of the (department-wide) testbed, with the implicit aim of being modularly extensible to wider (city-scale) scenarios.

WSNs can offer access to an unprecedented quality and quantity of information that can deeply change our ability to sense and control the environment. The fields of application of WSNs cover a wide variety:

- home automation (domotics) and energy management systems [3–6]: devoted to the monitoring and control of the environment of private homes for the comfort and security of their residents; this, especially in large buildings, may also include the management of their energy resources;
- assistive domotics [7], i.e., home automation for the elderly and disabled, where the general features of home automation are ancillary to those implied by regularly monitoring specific physiological and medical parameters of the residents;
- industrial automation [8]: aiming more specifically at the analysis and control of the environment (in terms of temperature, humidity, light, but also chemicals, vapors, radiation) in work places presenting critical issues of potential danger, such as, to cite a few, greenhouses, mechanical laboratories, chemical plants and refineries, foundries; this category also includes simpler issues such as the management and conservation of goods in large stores and warehouses;
- surveillance [9]: in terms of networks of cameras, microphones, access control devices, intrusion detection systems, and so forth. The integration and fusion of the information provided by single devices, using different technologies and from different physical points of view, allow a more complete (if not exhaustive) reconstruction of the whole scene of interest.

On a different scale, with an eye open toward the city-wide scale of interest or beyond:

- traffic monitoring and control [10–12]: such a sensor network would be exploited to monitor the vehicle flow, detect anomalous situations and alert the traffic police, identify and track specific vehicles or vehicle types; moreover, in case of traffic jams, it would provide information to support alternative route planning, and also some sort of city logistics strategy could be envisaged;
- pollution monitoring [13]: a sensor network distributed across the city would be an efficient tool to monitor pollution and presence of contaminants, both during normal city life and in case of emergency (e.g., for the detection of nuclear, chemical, or biological threats);
- surveillance [14] of open public places, such as parks, squares, streets, suburbs, or closed ones such as malls, schools, city halls, hospitals;
- real-time support for firemen and rescue squads [15] to locate themselves, and to navigate inside a building in case of emergency; moreover, this might include communicating the fireman position to external supervision centers, in order to improve coordinated search strategies;
- precision agriculture [16–18]: recently, the use of sensor networking technologies in farming activities is becoming more popular, in order to optimize fertilizer use, to choose appropriately the species to grow, and more in general to perform on crops the right operations at the right time;
- habitat and environmental monitoring [19–22]: surveillance of natural areas, such as natural parks, so as to favor the timely detection of events such as wildfires or floods, but also to collect data regarding the inhabitant populations of animals and plants; this category also includes the class of low-power weather monitoring applications, a good example of which is the Collaborative Network for Atmospheric Sensing (CNAS) project [23, 24].

The overall objective of the WISE-WAI project [25] is precisely to exploit the potential of WSNs by designing and evaluating a system architecture for a flexible, heterogeneous, and energy-efficient network, including the specification of applications. A number of experimental testbeds have been deployed in the past at various institutions (see, e.g., [24, 26–31]): the WISE-WAI project also aims at deploying a wireless sensor network testbed on a large scale, that will be employed to simulate deployments of a large number of nodes over a wide territory.

The project efforts will be spent at several design levels, and will be driven by the applications the network will be required to support. In the context of the WISE-WAI project [25], three classes of applications are of specific interest. *Location-based* applications are among the first and most popular applications of WSNs, since they can be employed for locating moving objects in buildings (e.g., warehouses, hospitals), tracking people inside areas of interest, as well as tracking movements in a battlefield. *Environmental monitoring* applications are also of primary importance, for ameliorating the quality of life through the definition of comfort criteria and the computation of related indices, to increase energy awareness and improve the efficient use of energy resources, as well as to guarantee safety and quality of the environment in work places. *Alarm event detection* is also of interest, especially in working or living conditions that can be subject to hazardous events (e.g., fire, leaks of gas or other poisonous substances, and so forth). The cited sample applications have been identified as of interest for the project because of the inherently different amount of traffic generated by each application type. For example, location-based tasks, that require the estimation of the position of one or more subjects, tend to generate a large, highly local traffic, as only the sensors in the proximity of the objects to be located interact for location estimation. Conversely, environmental monitoring usually generates lower, more uniform traffic, as typically all sensors are required to periodically report a measurement back to a collection station (sink). Finally, alarm events usually trigger high priority traffic over all sensors that are located in the proximity of the event, and require that this traffic be conveyed to the sink, perhaps removing the inherent redundancy of the data packets through some in-network aggregation technique [32].

The network structure required to support these traffic requirements must therefore be very flexible and capable to adapt to varying network conditions: e.g., local congestion can arise in case of long alarm events, and must be properly managed. With this target in mind, various MAC protocols are being considered for implementation in the network, with special regard to those that can offer energy savings and adaptation to local traffic requirements [33–35], or that can be integrated with routing as data is reported back to the sink [36].

While some portions of the network may be powered and programmed by means of a USB connection, other parts will be completely wireless, and thus need specific tools to be configured over the air. These tools will include efficient and error-resilient transmission schemes for broadcasting the software to be installed on the sensors, as well as an effective communication scheme to achieve fast reconfiguration of the nodes over multihop networks.

The applications will also be written with an eye open toward communication requirements: for instance, localization and tracking will be performed by communicating to a subset of the sensors available in the proximity of the object to localize, while environmental monitoring and alarm event detection will include specific functionalities that reduce the amount of transferred data through aggregation.

Finally the testbed will be organized in order to favor management tasks, as well as protocol design and test activities. The suite of protocols and applications written for the WISE-WAI testbed will then be made available to the public, so as to represent a suitable starting point to favor future WSN deployments of the same kind. The next section will delve into the organization of the WISE-WAI testbed, while the following sections will detail two of the network functionalities mentioned above, namely wireless reprogramming and localization/tracking.

## 2. Network Hardware and Software Architecture

In order to implement the above concepts, and provide a flexible and reconfigurable platform for testing algorithms and solutions for WSNs, we set up a large testbed including wireless nodes as well as networking devices that allow fast communication with the sensors (e.g., for reprogramming purposes). The resulting network will have in general medium density (i.e., the average number of neighbors within the coverage area of a given node is on the order of 10 to 20), but it should be noted that this can be tuned to some extent, by acting on the maximum transmit power of the sensor nodes. We deployed the testbed so that every node is connected to the network backbone through a Universal Serial Bus (USB) cable, which also provides power supply: this avoids battery wastage and continuous replacements during setup and test phases. During actual operations, however, communications take place only through the wireless channel. The USB backbone also provides a cheap and fast way to log data for debugging, of performing general management and of programming nodes as well; however, because the object of the project is to design and experiment wireless sensor networking on a large scale, efficient algorithms for reprogramming nodes wirelessly will also be deployed and tested, see Section 3.

As anticipated in Section 1, a WSN can serve many different applications, that in turn exhibit diverse requirements in terms of data transport performance, delivery delay, long-term operation (thus low energy consumption) and adaptability. For this reason, a most important design objective is that the network be scalable, easy to install, remotely manageable and reachable in case maintenance operations should be carried out. The networking backbone plays a key role in all these aspects.

We have chosen a hierarchical organization, whereby all sensors are connected, via USB hubs, to tiny embedded computers that act as Node Cluster Gateways (NCGs). The NCGs are in charge of interacting with the nodes both in the upstream (node-to-gateway) direction, e.g., for reporting debug and log messages, and in the downstream (gateway-to-node) direction, e.g., to reprogram, reset, and power up or down the nodes, or to adapt the node behavior on the fly as needed by ongoing measurement tasks. The gateways are connected via an Ethernet backbone to a local remote access gateway, that manages local communications within specific portions of the network. All remote access gateways are finally connected to a central server through Virtual Private Network (VPN) tunnels. The server is the main point of access for the communication to, and the management of, the whole WSN. It is planned that, at the end of the project, the research community will be given access to the measurement history available on the server storage.

The aforementioned architecture is scalable, easy to replicate in case the network needs to be extended, and in addition its components are easily reached and replaced for maintenance. In particular, it should be noted that the full USB 2.0-compliant hubs employed in the testbed allow a sort of hard sensor reset, which is accomplished by powering off the port to which the sensor is connected. Thanks to this function, the sensors need not be manually disconnected, in case they do not respond to software reset commands. For all these reasons, dividing the network into smaller subsets that are managed through gateways, while using embedded PCs and USB hubs to ease remote control, provides a better solution. In particular, NCGs are a key component of our network hierarchy. They are small computers of size 15 cm × 15 cm, bearing limited power supply requirements, which can be supported through the Power-over-Ethernet (PoE) standard. Each NCG hosts the connection to sensors via USB hubs.

Finally, the wireless embedded sensors we chose for our testbed are the TelosB nodes [37]. This choice has been made for two reasons: firstly, the TelosB platform is undergoing widespread use around the world and therefore it is constantly supported and upgraded; secondly, it will be easier to integrate this network with two already deployed testbeds, namely SensNet and SignetLab, that have been set up inside two rooms of the department using the same sensor nodes. The TelosB sensors are low-power wireless nodes used in WSNs, and come equipped with temperature, humidity and light sensors. They can be directly connected to other devices through an embedded USB port. A wireless connection is also directly available through an implementation of the ZigBee protocol stack. TelosB nodes use the CC2420 radio chip for ZigBee-compliant communications in the 2.4 GHz band, in accordance to the IEEE 802.15.4 standard. Their maximum transmission power is 1 mW within the 2400-2480 MHz bandwidth. Their transmission rate of 250 kbps is foreseen to be enough to support all wireless sensor network applications that we consider in the WISE-WAI project.

### 2.1. Web-Based Testbed Interface

**Figure 1.**
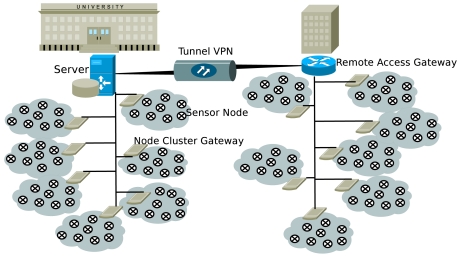
Campus-wide system architecture.

To make the setup of experiments and node programming easier, we have deployed an HTTP-based interface that can be reached, for the time being, only from the Department Intranet. It is envisioned that a form of limited access will be granted to registered web users in the near future. The HTTP interface masks the network structure and allows to perform the main operations required for network usage, such as the installation of certain applications on the nodes as well as power up or shutdown functions. It should be noted that the presence of the NCGs is completely hidden to the users, who perceive instead the network as a whole (or in other words, as though there were only one comprehensive cloud in Figure 1).

**Figure 4.**
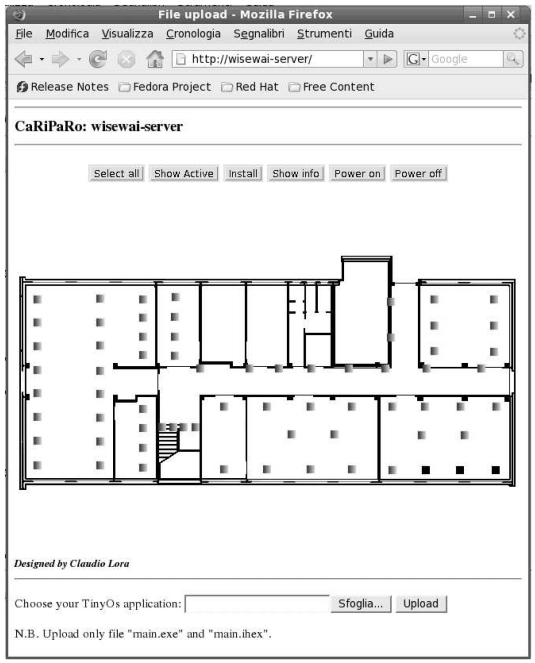
Web-based graphic interface for the WISE-WAI testbed.

The graphical interface is shown in Figure 4. The map of the nodes is displayed and contextualized by superimposing it on the building map. Through the interface, we can select one or more nodes (shown in darker grey at the bottom-right corner of Figure 4) and choose a function such as *Power on* or *Power off*. A *Show active* button highlights the nodes that are currently switched on. The *Show info* command pops up additional information such as the node location, the loaded application, and the Ethernet port address associated to the node: this information is necessary for application development and management purposes. Before reprogramming any set of sensors, the application to be installed must be copied on the server (which will also keep a local log of the applications that have been uploaded) using the *Upload* button, before clicking on *Install*, which actually loads the application.

This section has covered the wired, USB-powered portion of the WISE-WAI testbed. The final testbed will also feature completely wireless portions, which will be installed to cover rooms where cabling cannot be laid, as well as to increase the coverage of specific sections of the buildings, as required. These sensors will be battery-operated through high capacity 2600 mAh packs. Preliminary tests on this setup have shown that a continuously transmitting node can operate for nearly 4 weeks before exhausting its energy supply. We are working to extend this operational time from 1 to 6 months, by means of efficient energy saving procedures at the networking level.

The following sections are dedicated to describing some applications that have been deployed so far on the WISE-WAI testbed. The first, in the next section, is actually a service that provides a wireless node reprogramming function, which allows to reprogram the nodes which are not connected to the network backbone. Section 4. will then detail the localization and tracking applications, as well as the related problem of identifying the radio channel parameters, in order to provide good ranging performance.

## 3. A Fundamental Network Service: Wireless Reprogramming Using SYNAPSE

Wireless network reprogramming represents an essential functionality for Wireless Sensor Networks (WSNs). With it, a user can install new software on network nodes, load new parameters for a given protocol, add functionalities to the WSN, and so forth. Re-programming/re-tasking nodes wirelessly is of particular importance due to the inherent multi-hop nature of WSNs and to the fact that these networks in some cases may be expected to host hundreds or even thousands of nodes. Given that, reprogramming must be done indeed through automatic and yet efficient procedures. Also, as WSNs in this project are exploited as a part of a more complex system for environmental (e.g., urban) monitoring, reprogramming, dynamic configuration, re-tasking and network management are fundamental features.

Several re-programming protocols for WSNs were proposed in the last few years [38–43], the best known being Deluge [40] and MNP [41]. Deluge disseminates the code in multi-hop environments exploiting an epidemic routing algorithm, which uses a three-way handshake based on advertisement (ADV), request (REQ) and actual code (CODE) transfer. Many issues are tackled and solved by this protocol. First, the program image is split into smaller parts, referred to as *pages*, which are then disseminated through an epidemic algorithm to WSN nodes. These parts are then independently disseminated using a NACK-based ARQ protocol. The code is transmitted, page by page, via broadcast, and pipelining is implemented. Pipelining allows a node that correctly receives a page from a neighboring sensor to promptly start the dissemination of this page to the next hop. In other words, it increases the degree of concurrency in the network during data dissemination. The randomization of the transmission of the advertisement within predetermined time windows, as well as advertisement suppression, are implemented to reduce congestion in the propagation of the code through multiple hops. MNP [41] has many features in common with Deluge. In addition, it implements special algorithms to reduce the problems due to collisions and hidden terminals. This is achieved through a distributed priority assignment so that, in a neighborhood, there is at most one sender transmitting the program at any given time. The sender election is greedy and distributed, i.e., there is no need to know the topology in advance. In MNP the senders with a higher number of potential receivers are assigned higher priority and sleeping modes are also used to reduce energy consumption; a sender can go to sleep when a neighbor with higher priority has data to send.

While much work has been done already, data dissemination in multi-hop environments still has some performance problems. These are due to the large number of nodes that may potentially interact and collide (also known as hidden terminal problem). As a result, nodes in the center of the network get longer programming times and tend to consume more energy with respect to those placed at the network edges. (See [40] for experimental results showing this.) Our activity focused on this problem and, in particular, on the design of advanced link layer solutions to mitigate the performance issues that arise in dense multi-hop scenarios.

**Figure 5.**
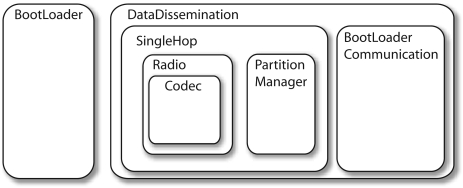
SYNAPSE's software architecture. Reproduced with permission from IEEE [44].

**Figure 6.**
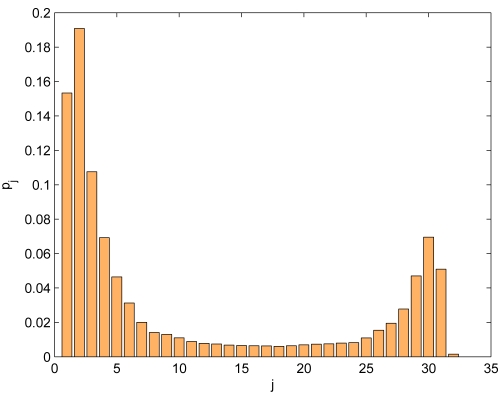
SYNAPSE's encoding distribution *p*(*j*), returning the probability that *j* original packets out of a source *page* of *K* = 32 packets are combined to generate an encoding packet. Reproduced with permission from IEEE [44].

**Figure 7.**
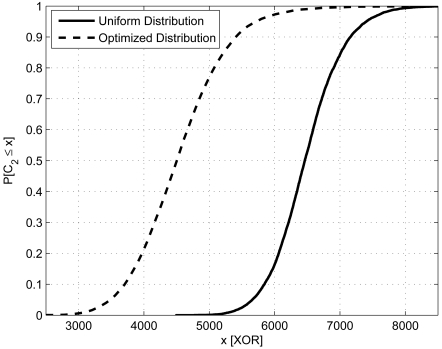
Decoding cost comparison. Probability that the number of XORs needed for decoding a program *page* is at most *x*. Reproduced with permission from IEEE [44].

**Figure 8.**
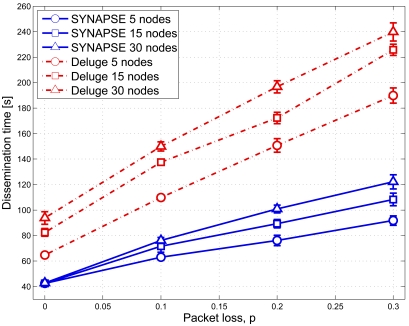
Program dissemination time as a function of the packet error rate *p* for an application of 27, 100 bytes. Reproduced with permission from IEEE [44].

We designed a novel network re-programming scheme for WSNs, which we called SYNAPSE (see Figure 5 for a sketch of its architecture). SYNAPSE features various modules dealing with radio transmission (Radio), the dissemination of the program image in single- (SingleHop) as well as multi-hop (DataDissemination) scenarios, memory management (PartitionManager) and device/application status control (BootLoader and BootLoaderCommunication). In addition, there is a module dealing with link layer error control and coding (Codec) which is the core of SYNAPSE's data dissemination algorithm. We designed it using Fountain Codes [45], which are recent and very efficient codes for sending data to multiple senders, as in our case. Fountain Codes, however, could not be applied to our problem off-the-shelf, but substantial work had to be done in order to adapt them to the specific constraints of sensor devices. Without delving too much into the specific details of our approach, suffice it to say that we had to design encoding distributions able to work with small program and packet sizes (which are typical of WSNs) and which retain the good properties (in terms of throughput efficiency and error correction capability) of the longer codes proposed in the literature [45, 46]. We did this through an original genetic optimization approach. The optimized degree distribution (see Figure 6), which we used for encoding and distributing program images to WSN nodes, leads to sparse decoding matrices. These are good as their inversion (and hence the recovery of the original data) can be done at low computational cost, in accordance with our system requirements. In Figure 7 we show the cumulative distribution function of the decoding complexity, expressed in terms of number of XORs that we need to recover the original data at the receivers. From this plot it can be seen that our optimized distribution requires a substantially smaller number of operations per *page*. Figure 8 shows experimental results about the network re-programming time for SYNAPSE and Deluge. In this graph we show different curves varying the number of nodes, while the abscissa represents the packet error rate (which was emulated through a software module to allow a precise characterization of the protocol behavior).

A demonstration of the SYNAPSE re-programming suite was provided at the SenSys 2007 conference [47]. More detail on SYNAPSE can be found in [44]. The source code of the current version of SYNAPSE can be downloaded from [48].

The SYNAPSE suite is currently being updated with several functions with the purpose to go beyond pure reprogramming and to obtain a fully functional *reprogramming* and *management* framework for WSNs. Some of these features are:

- Implementation of pipelining techniques for our fountain-code-based dissemination protocol. This will allow improved performance in distributed, densely populated and ultimately multi-hop networks.
- Implementation of tools for data/node management such as: 1) acquiring the memory status of selected nodes prior to or after reprogramming (in both single- as well as multi-hop networks), 2) sending commands to sensor nodes in order to, e.g., reset them, load and execute a new application, handle memory utilization, get the energy status of sensor nodes, etc.
- Integrate SYNAPSE (and the whole WSN) with more complex networking scenarios where sensors can be either controlled by nodes placed within the fixed Internet or by mobile nodes through a different radio technology (i.e., IEEE 802.11g). This entails the integration of the WSN system with intelligent gateways which will translate WSN messages into IP packets through, e.g., IP tunneling (with de-tunneling at the controller).

## 4. A Typical Application: Localization and Target Tracking

One popular application for WSNs is localization, tracking and navigation of moving objects in indoor environment using the RF signal strength. To fix ideas, say that we are interested in designing a realtime system that can help fireman rescue squads locate themselves and navigate inside a building during emergencies. To achieve this objective, we propose to deploy a static wireless sensor network whose nodes are placed at known positions. Each fireman is provided a pocket-PC, or similar device, attached to a mobile node which can communicate with the static network. The position of firemen is estimated only by using the radio signals, i.e., through the Received Signal Strength Indicator (RSSI) and Link Quality Indicator (LQI) provided by a standard IEEE 802.15.4 [49] radio chip, without resorting to any other special sensor or devices such as infrared motion sensors, ultrasound, or directional antennas.

The network of fixed and mobile nodes implements a distributed intelligence system. In particular, we chose to leave the processing required for the localization of the fireman to the pocket-PC rather than to the static network, since the former bears greater computational capabilities, and can thus run localization algorithms faster. This solution is also more scalable, since the nodes of the static network simply have to transmit their own location (or node ID, if pocket-PCs have a stored map which associates node IDs to locations) regardless of the number of firemen. Each pocket-PC computes it own position and displays it on a screen with a map of the environment, similar to commercial GPS-based navigators. The position is also retransmitted to the static network, which routes it back to a gateway, and from there to the firemen coordination center. This allows to support centralized coordination of the firemen squad efforts, as well as fast firemen localization in case of accidents. Moreover, the coordination center can plan a set of way-points for each fireman that are retransmitted back to the pocket-PC for navigation.

Different approaches to indoor localization based on acoustic time of flight [50], directional antennas [51], and cameras [52] have been proposed to date. However, RSSI-based localization systems are much more popular, since most radio chips for WSNs provide RSSI at no extra hardware cost. In an ideal medium with an ideal antenna, there should be an information-preserving correspondence between each RSSI value and the relative distance between two nodes; however, in an indoor environment, multipath fading, reflections, diffraction, interference, and environmental dynamics highly affect this relation. Although the localization accuracy is poorer than achieved by using non-RSSI localization systems, this accuracy is in fact sufficient in many applications such as the firemen support we address here. Most of the algorithms coping with the high variability of RSSI measurements can be grouped into two distinct classes that we refer to as *RSSI map-based* localization and *RSSI range-based* localization. The former strategy consists in mapping the signal strength footprints as generated by each static wireless node, and then estimate the most likely location given a set of new RSSI signatures generated by the moving target. In particular, the RSSI map takes into account the location of the static nodes as well as the topology and morphology of the environment, including walls and static objects. The most popular tracking systems in this class are the RADAR [53] and MoteTrack [54] tracking systems. RSSI range-based algorithms, instead, first try to estimate the relative distance of the moving node from each of the static nodes, and then triangulate the moving node location using a geometric approach [55], similar to GPS. The advantage of this strategy is that it does not require any a priori detailed RSSI-map of the environment, however it suffers from greater inaccuracy in cluttered environments, if the node density is not sufficiently high. It is important to remark that the localization error is not the only important factor that needs to be considered when designing an appropriate tracking system. Other important aspects such as the number of nodes to achieve a desired localization error, the computational requirements necessary to run the proposed algorithms in real-time, the installation and maintenance costs, the system lifetime, robustness to dropped packets and node failures, and scalability must also be taken into account.

Based on these considerations we opted for a range-based localization system due to its better scalability, its low installation and maintenance costs, and its high robustness to link and node failures. However, as compared to current implementations of range-based localization systems [55] we added three main features that help mitigate the effects of non-isotropic signal strength decay in indoor environments. The first feature is a distributed self-calibration algorithm that removes possible RSSI offsets at the receiver stage, thus improving the estimation of the relative distance between two nodes. The second feature is a real-time distributed identification algorithm for the RSSI channel parameters which allows each node to set the correct values based on measured RSSI from its neighbors after installation. The third feature is the use of a Kalman Filter for improving the current target location based on the current RSSI measurements and the past target estimated location. In order to test the validity of our solution, we implemented and tested our tracking system in a cluttered and noisy indoor environment. In the remainder of this section, we briefly describe the algorithms we employ, the network architecture and some of our experimental results.

### 4.1. A Preliminary Step: Channel Modeling and Analysis

As anticipated in the introduction to this Section, we recall that the received signal power is a very noisy piece of information due to many factors, including: the transmitter and receiver offsets due to small differences in the hardware components that arise during factory construction processes; the high variance in channel behavior because of multipath fading; the estimation of radio propagation parameters, which can affect the accuracy of ranging operations.

In order to find a good model for the analysis of the channel, we consider here a simple path loss channel model, in which a generic node *i*, placed at distance *d_i_* from the transmitter, receives a signal with power *P_i_* (in dBm) given by

(1) $$P_{i}=P_{\text{loss}}(d_{i})+\Psi_{i}+w_{i}(t),$$

where

(2) $$P_{\text{loss}}(d_{i})=P_{Tx}+K-10\eta \log_{10}\left[\frac{d_{i}}{d_{0}}\right].$$

In (2), *P_Tx_* is the nominal transmission power in dBm, *K* is a constant that depends on the environment, *d*_0_ is a reference distance for the antenna far field, and *η* is the path loss coefficient. The term Ψ*_i_* denotes the random attenuation due to shadowing, while *w_i_*(*t*) accounts for the fast fading effect [56]. Typically, shadowing is almost constant over long time periods, while fast fading shows rapid fluctuations, so that packets received in different time epochs likely experience equal shadowing, but almost independent fading effects.

Assuming that the transmitter and receiver of a packet do not move, the fast fading term in (1) can be averaged out by considering multiple channel readings by the same receiver. Thus the channel model becomes as follows:

(3) $$P_{i}≃P_{Tx}+K-10\eta \log_{10}\left[\frac{d_{i}}{d_{0}}\right]+\Psi_{i}.$$

Medium–scale shadowing, however, cannot be easily removed when both transmitter and receiver are stationary. The statistical distribution of this factor is generally assumed to be Gaussian, with zero mean and standard deviation *σ*_Ψ_*i*__ whose value ranges from 4 up to 12 depending on the characteristics of the environment [56]. We remark that in this study we assumed the shadowing terms to be independent and identically distributed.

In order to determine the path loss model parameters *K* and *η*, given *d*_0_, as in (2), we have picked one of the possible radio channels where the TelosB nodes in our testbed can operate, and have then averaged a number of received power samples in order to remove the fast fading effects. We have used the least square approach for identifying the channel parameters *K, η* and *σ*_Ψ_*i*__ in (3), and finally obtained *K*≃ −25, *η* ≃ 2.2 and *σ*_Ψ_*i*__ ≃7.5dB.

In order to improve the accuracy of the parameter identification, we then exploited the capability of TelosB sensors to communicate over different channels. In more detail, we perform a separate measurement campaign over each of the available transmit channels, i.e., by switching the carrier frequency in steps of 5 MHz, from 2.405 GHz to 2.480 GHz. We remark that in our tables and graphs, the 2.415 GHz, 2.425 GHz and 2.430 GHz frequencies are not taken into account due to the interference caused by the Department's WiFi network. We then considered an RSSI measurement for a pair of nodes as the average of all received powers over all channels, as in (4):

(4) $$P_{i,j}=\frac{1}{N_{ch}}\sum_{ch=1}^{N_{ch}}\frac{1}{N_{\text{meas}}}\sum_{n=1}^{N_{\text{meas}}}P_{i,j}^{n,ch},$$

where *N_ch_* is the number of channels considered and *N_meas_* is the number of RSSI measurements performed over the same channels (*N_meas_* = 10 here).

By studying the multi-channel behavior we observe two results: *i*) the parameters estimated for different channels are very similar (see Table 1), and *ii*) the reliability of RSSI measurements improves considerably, after the estimation of channel parameters using averages obtained over all channels.

**Figure 10.**
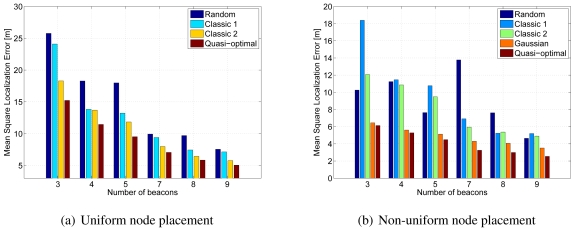
OMSE for different beacon deployment schemes with RSSI-based localization.

In more detail, by considering any of the 13 different channels separately, we can notice that each estimation leads to similar values of *K, η* and $\sigma_{\Psi_{i}}^{2}$. Therefore the choice of a particular channel does not affect the output of parameter identification, thus of the reliability of RSSI as used for ranging purposes. On the contrary, by using the RSSI information from every channel, we observe that the parameters *η* and *K* do not change very much with respect to the single-channel campaign, whereas the value of the variance $\sigma_{\Psi_{i}}^{2}$ is smaller. While this indicates that *K* and *η* are estimated correctly even using a single channel, the reduction of $\sigma_{\Psi_{i}}^{2}$ implies a better reliability of the RSSI measurements and therefore a better performance experienced by applications that infer distance through RSSI, such as localization and tracking. Two examples of channel models obtained through measurements over one channel and over all channels can be seen in Figures 10(a) and 10(b), respectively. The figures also convey evidence of the greater variance of RSSI samples taken over a single channel.

At this point, we make one step forward by recalling that, as anticipated before, range-based localization algorithms imply the presence of some nodes, called *beacons* (also known as *anchors* or *landmarks*), which are aware of their geographical positions. Beacons are required to periodically broadcast their coordinates, in order to let the other nodes in the network, referred to as *stray* nodes, infer their own position by means of some estimation technique.

In the literature, the localization problem has been primarily tackled from the perspective of the stray nodes, thus leading to the design of schemes and hardware that could reduce the positioning error from the signals received from beacons. In our work, instead, we turn our attention to the impact of the *beacon positioning* on the performance of the localization algorithms. We investigate how the mean square error of the position estimation is affected by the number and location of the beacons in the area.

We consider an indoor square area 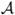 containing *M* beacons. Let:

(5) $$\xi_{i}=(\begin{array}{ll} x_{i} & y_{i} \end{array})\;\xi \Xi =\left(\begin{array}{c} \xi_{1}\\ \xi_{2}\\ \vdots \\ \xi_{M} \end{array}\right)$$

denote the geographical coordinates of the *i*th beacon, for *i* = 1, 2,…, *M*, and the *M* × 2 matrix of all beacon coordinates, respectively. The stray (i.e., non localized) node can be located at any position **s** = (*x, y*) in the area, according to a given two-dimensional probability density function (pdf) *f*(**s**) : 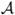 → ℝ, for which it clearly holds ∬_
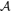
_ *f*(**s**)*∂***s** = 1.

We define the ranging estimation using well known model for both RSSI [56] and ToA [57] techniques. Finally we estimate the node's position using the Maximum Likelihood algorithm. Accordingly, the mean square estimation errors is *ε*(**s**) = E [‖**s** − **ŝ**‖^2^], where **ŝ** is the estimated position of the node. Averaging over the position of the stray node, we finally get the *overall mean square error* (OMSE) of the position estimation:

(6) $$\varepsilon (\Xi )=\iint_{(x,y)\in A}\varepsilon (x,y)\;f(x,y)\text{dxdy}$$

where we have made it explicit in the notation that the OMSE depends on the beacon position matrix B. Our aim is to determine the matrix Ξ *_opt_* which minimizes (6), i.e.,

(7) $$\begin{array}{ccc} \Xi_{\text{opt}}=\arg & \begin{array}{c} \underset{\xi_{i}\in A}{\min}\\ i=1,\ldots ,M \end{array} & \varepsilon (\Xi ) \end{array}$$

In order to understand the impact of the beacons position on the OMSE index, we considered three positioning strategies, which we named *random, classic*, and *(quasi)-optimal*. The random strategy simply consists in placing the beacons at random in the area. We include this as a limit case in our study, in order to have a reference value that allows to better appreciate the performance gain obtained from an accurate planning of the beacons' position. In the classic strategy, the beacon placement is driven by a common-sense criterion, according to which beacons should be preferably placed along the perimeter, specifically in the corners and the center of the edges delimiting the area, or be equally spaced within the area. Finally, the quasi-optimal strategy corresponds to finding the solution to (7) by quantizing the position of the beacons in order to reduce the complexity of the problem.

Results have been obtained by considering an area 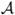 of 10 × 10 square meters, divided into *N*^2^ = 100 cells of 1 × 1 square meters each. Figure 10 shows the OMSE for different beacon positioning strategies and for different numbers of beacons. We can observe that our OMSE-minimizing scheme increases the accuracy of the position estimation up to 40% especially if nodes have a non-uniform distribution.

### 4.2. Real-Time Sensor Calibration and Channel Parameter Identification

Modeling the radio channel in indoor environments is rather complex due to reflections, interference, multiple paths and time-varying and non-isotropic propagation effects. As mentioned above, the map-based localization approaches try to learn this model by performing many measurements by positioning the unknown node in some predetermined fixed location. Differently, we use an isotropic RSSI model with polynomial decay as a function of the relative node distance. More precisely we rewrite the channel model of RSSI received by node *i* from node *j* defined in (3) as follows:

(8) $$P_{rx}^{ij}(t)=\beta -10\eta \log_{10}(d_{ij})+v_{ij}+w_{ij}(t)+o_{i}$$

where we set *d*_0_ = 1,Ψ*_i_* = *v_ij_* + *o_i_, P_Tx_* + *K* = *β*, while *d_ij_* = ‖*ξ_i_* − *ξ_j_*‖ is the relative node distance, *ξ* = (*x, y*) is the location of the node, *β* represents the radio receiver gain for constant transmission power at a nominal distance of *d_ij_* = 1 m, *η* is the loss factor (*η* ≃ 2 in a free-space propagation setting), *w_ij_*(*t*) represents zero-mean white noise, and *v_ij_* is a disturbance that depends only on nodes location and is due to slow fading, and *o_i_* is the RSSI offset due to fabrication imperfections of the receiver stage. The fast fading term *w_ij_*(*t*) can be removed by averaging *T* consecutive measurements if the nodes do not move, i.e.,

(9) $${\bar{P}}_{rx}^{ij}=\frac{1}{T}\sum_{t=1}^{T}P_{rx}^{ij}(t)\approx \beta -10\eta \log_{10}(d_{ij})+v_{ij}+o_{i}$$

As shown below, the RSSI offset *o_i_* can be estimated, i.e., we can compute *ô_i_* such that *ô_i_* ≈ *o_i_*. Therefore, the calibrated averaged RSSI can be obtained as follows:

(10) $${\hat{P}}_{rx}^{ij}={\bar{P}}_{rx}^{ij}-{\hat{o}}_{i}=[1-10\log_{10}(d_{ij})]\left[\begin{array}{c} \beta \\ \eta \end{array}\right]+v_{ij}=h(d_{ij})+v_{ij}$$

The slow fading term *v_ij_* depends nonlinearly on the nodes location but it is symmetric, i.e. *v_ij_* = *v_ji_*, and we are going to treat it as an unavoidable measurement disturbance, although, in principle, it could be estimated using map-based techniques. It is important to remark that the parameters *η* and *β* are highly variable among different environments such as warehouses, residential buildings and laboratories, and therefore cannot be determined a priori but rather they should be estimated on-site. Below, we will show how to perform the identification of the parameters (*β, η*), and will especially focus on doing so using real-time distributed algorithms.

Both the offset calibration algorithm and the parameter identification rely on popular distributed optimization algorithms known as *average consensus algorithms* [58, 59], which are particularly suitable for networks where an agent cannot communicate to all other agents, but only to a small number of neighbors within radio coverage, which is the typical scenarios in a WSN. In particular, these algorithms allow to compute the average of local variables stored in each agent. More precisely, the network is modeled as a graph 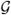 = ( 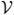, 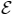), where the set of vertices 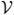 = [*1*,…,*N*] represents the agents, and the set of edges 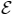 represents the communication links. The set of neighbors of a node *i* is defined as 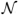
*_i_* = {*j* ∈ 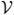 ∣ (*i, j*) ∈ 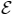}. Suppose that each agent *i* stores a variable *z_i_*, and would like to compute its average, i.e., $\bar{z}=\frac{1}{N}\sum_{i=1}^{N}z_{i}$. One strategy would be to collect all variables *z_i_* at one agent, then compute the average, and finally redistribute it to all agents, however this approach is not robust to node failure and requires global coordination among all agents. An alternative strategy is based on a distributed iterative algorithm which uses only local information exchange and converges to the desired value. This can be performed as follows:

(11) $$x_{i}(0)=z_{i}$$

(12) $$x_{i}(k+1)=q_{ii}x_{i}(k)+\sum_{j\in N_{i}}q_{ij}x_{j}(k)$$

where *k* is the iteration step and *q_ij_* = [*Q*]*_ij_* is the (*i, j*) entry of the matrix *Q* ∈ ℝ*^N^*^×^*^N^* which represents the algorithm parameters. It can be shown that under mild conditions, such as for example *q_ij_* ≥ 0, Σ*_i_* [*Q*]*_ij_* = 1, Σ*_j_*[*Q*]*_ij_* = 1, and the graph 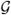 is connected, then

$$\underset{k\to \infty }{\lim}x_{i}(k)=\frac{1}{N}\sum_{i=1}^{N}x_{i}(0)=\frac{1}{N}\sum_{i=1}^{N}z_{i}=\bar{z},\;\forall i$$

In practice, the previous algorithm can be generalized to time-varying matrices *Q*(*t*) and also to randomized communication. A detailed discussion is beyond the scope of this work and we address the interested reader to [58, 59] and the references therein. For what concerns this work, the bottom line is that simple local linear updates of local variables allow to compute global averages, which is the foundation of the proposed sensor calibration and parameter identification algorithms.

As mentioned above, the receiver stage of the radio chip which provides the reading of the RSSI suffers from random variations from the actual value, thus providing RSSI measurements that can be off by 2-4 dB. This offset *o_i_* can degrade the estimation of the relative node distance and therefore of the overall localization accuracy of the moving target. In fact, an offset of 2 dB can result in an estimation error of 40 cm at a nominal distance of 1 m and of almost 3 m at a nominal distance of 10 m. Ideally, we would like to compute *ô_i_* = *o_i_*, however this is not possible unless we have a precise sensor to measure *o_i_*. However, since the offsets *o_i_* are likely to be independently distributed around the nominal value of *o* = 0, we can argue that $\frac{1}{N}\sum_{i=1}^{N}o_{i}=\bar{o}\approx 0$ if the number of nodes *N* in the network is sufficiently large. Therefore, we might opt for computing *ô_i_* such that *o_i_*−*ô_i_* = *ō*. This indeed can be re-framed as a consensus problem by setting *z_i_* = *o_i_* and *x_i_*(*k*) = *o_i_* − *ô_i_*(*k*) [60], which gives rise to the following iterative algorithm:

(13) $$\{\begin{array}{l} \begin{array}{cc} {\hat{o}}_{i}(0)=0 & \forall i\in N=1,\ldots ,N \end{array}\\ {\hat{o}}_{i}(k+1)={\hat{o}}_{i}(k)+\sum_{j\in N_{i}}q_{ij}({\bar{P}}_{rx}^{ij}-{\bar{P}}_{rx}^{ij}+{\hat{o}}_{j}(k)-{\hat{o}}_{i}(k)) \end{array}$$

where we used the fact that *q_ii_* = Σ_*j*∈ 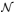
_*i*__ *q_ij_, v_ij_* = *v_ji_* and $o_{i}-o_{j}={\bar{P}}_{rx}^{ij}-{\bar{P}}_{rx}^{ji}$. Based on the considerations given above we have that

$$\underset{k\to \infty }{\lim}{\hat{o}}_{i}(k)=o_{i}-\bar{o}\approx o_{i}$$

After the RSSI sensor calibration phase, the next important step in the localization is to identify the RSSI model parameters (*β, η*) defined in (10). Each node *i* of the fixed wireless network knows its own position *ξ_i_* and RSSI offset estimate *ô_i_*, and can communicate them to its neighbors 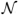
*_i_*, therefore each node can compute ${\hat{P}}_{rx}^{ij}$ and the corresponding *d_ij_* = ‖*ξ_i_* − *ξ_j_*‖. The problem of estimating (*β, η*) can be recast as a least square estimation problem where the objective is to find

(14) $$(\hat{\beta },\hat{\eta })=\arg\min_{\beta ,\eta }\sum_{i=1}^{N}\sum_{j\in N_{i}}({\hat{P}}_{rx}^{ij}-{\left(\beta -10\eta \log_{10}(d_{ij})\right)}^{2}=\arg\min_{x}{\left\|Ax-b\right\|}^{2}={\left(A^{T}A\right)}^{-1}A^{T}b=\hat{x}$$

where *x* = [*β η*]*^T^* is the column vector of the unknown parameters, $b={\left[\cdots {\hat{P}}_{rx}^{ij}\cdots \right]}^{T}$ is the column vector obtained by stacking the offset-compensated RSSI ${\hat{P}}_{rx}^{ij}$ measured by all nodes, and *A^T^* = [⋯*a_ij_*⋯] is the matrix obtained by stacking the corresponding vectors *α_ij_* = [1 − 10log_10_(*d_ij_*)]*^T^*. Although the solution of this problem requires to collect all data in some centralized location and then send back the computed parameters to all nodes, it is possible to recast it as an average consensus problem [60] by noting that

$${\left(A^{T}A\right)}^{-1}A^{T}b={\left(\sum_{i=1}^{N}\sum_{j\in N_{i}}a_{ij}a_{ij}^{T}\right)}^{-1}(\sum_{i=1}^{N}\sum_{j\in N_{i}}{\hat{P}}_{rx}^{ij})={\left(\frac{1}{N}\sum_{i=1}^{N}\sum_{j\in N_{i}}a_{ij}a_{ij}^{T}\right)}^{-1}(\frac{1}{N}\sum_{i=1}^{N}\sum_{j\in N_{i}}{\hat{P}}_{rx}^{ij})$$

which can be computed by running two average consensus algorithms in parallel by setting as initial conditions at node $i\;{\text{x}}_{i}^{A}(0)=\sum_{j\in N_{i}}\;a_{ij}a_{ij}^{T}$ and ${\text{x}}_{i}^{b}(0)=\sum_{j\in N_{i}}\;{\hat{P}}_{rx}^{ij}$ so that $\lim_{k\to \infty }{\left({\text{x}}_{i}^{A}(k)\right)}^{-1}{\text{x}}_{i}^{b}(k)=\hat{x}$.

This algorithm can be run on-site, and its convergence rate depends on the number of nodes and edges in the network graph. Moreover, the algorithm can be periodically re-run in order to adapt to environmental changes due, for example, to furniture repositioning or different use of the building between day and night.

### 4.3. Localization and Tracking

Once the model parameters (*β, η*) are identified, one typical range-based approach to estimate the location *ξ* of an unknown moving node is to measure the RSSI from a number of *N* fixed nodes whose positions *ξ_i_, i* = 1,*…, N* are known, then use the previous RSSI model to estimate the relative distance by inverting the model, i.e. ${\hat{d}}_{i\ell }=h^{-1}({\hat{P}}_{rx}^{i\ell })=10^{\frac{\beta -{\hat{P}}_{rx}^{i\ell }}{10\eta }}$, and finally triangulate the position of the unknown node *ℓ* based on its relative distances *d̂_i_*_ℓ_ from the known node positions *ξ_i_* [55]. Differently, in this work we propose the use of an Extended Kalman Filter (EKF) which has the advantage of providing smoother estimates since it exploits memory of past location estimates of the moving object. The EKF is based on a simple model for the dynamics of the moving object, namely a random walk along each of the two directions (*x, y*), and on the measurement model given by (10). More precisely the dynamical model is given by:

(15) $$\xi (k+1)=\left[\begin{array}{cc} 1 & 0\\ 0 & 1 \end{array}\right]\;\xi (k)+\text{w}(k)$$

(16) $$\psi (k)=\left[\begin{array}{c} {\hat{P}}^{1}(k)\\ \vdots \\ {\hat{P}}^{N}(k) \end{array}\right]=\text{h}(\xi (k))+\text{v}(k)=\left[\begin{array}{c} h\left(\left\|\xi (k)-\xi_{1}\right\|\right)\\ \cdots \\ h\left(\left\|\xi (k)-\xi_{N}\right\|\right) \end{array}\right]+\text{v}(k)$$

where *ξ*(*k*) = [*x*(*k*), *y*(*k*)]*^T^* ∈ ℝ^2^ is the position of the moving node ℓ at time *k, ψ*(*k*) is the vector of the RSSI measurements received by the moving node from the *N* fixed nodes, and the function *h*(*d*) = *β* − *10η* log_10_(*d*) was defined in (10). Note that, if the RSSI measurement from a generic node *i* is not received at time *k*, then the corresponding *P̂^i^* (*k*) is removed from the vector *φ*(*k*). The Extended Kalman Filter is based on the measurement process, linearized around the current location estimate *ξ̂*(*k*), i.e.,

$${\psi_{\text{lin}}(k)=H(k)\xi (k)+\text{v}(k),\;H(k)=\frac{\partial \text{h}}{\partial \xi }|}_{\xi =\hat{\xi }(k)}$$

and on the assumption that **w**(*k*) and **v**(*k*) are uncorrelated, zero mean white noises, with covariance 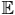
[**w**(*k*)**w**(*k*)*^T^*] = *Q* and 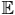
[**v**(*k*)**v**(*k*)*^T^*] = *R*.

The details of the algorithm and some interesting variations are beyond the scope of this paper and the interested reader is referred to [61]. However, some experimental results are shown in the next subsection.

### 4.4. System Implementation and Experiments

**Figure 11.**
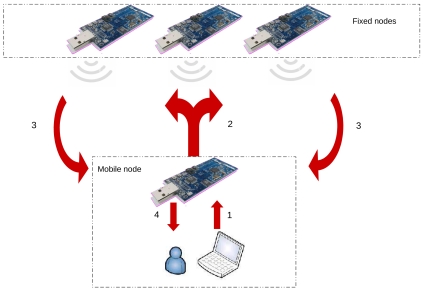
Initialization of tracking procedures. A start activation message (1) is broadcast to the network (2). The received data message (3) is (as far as the localization application is concerned) uniquely used to estimate the RSSI value (4), that is then processed to obtain the position estimation.

The localization and tracking system setup is based on the deployment of a fixed network of *N* nodes placed at known locations, which serve as reference anchor nodes for mobile nodes, that are free to move in the monitored environment. All communications among the agents occur via wireless links. In addition, the mobile node is equipped with some additional computational power (a portable PC system, or a palm PC device) to perform the location estimation algorithms: in this respect, the mobile node actually receives messages from the fixed nodes to estimate the RSSI values, which are then transmitted via a USB link to the PC system for the processing algorithms. The sequence of communication messages is visually described in Figure 11.

In more detail, after the user activates the localization system (step 1), the mobile node proceeds by sending periodic messages every 250 ms to the fixed node network in order to awake it and keep it active (step 2). The fixed nodes remain in the active state for further 250 ms, and in this state they start sending data messages (step 3) containing their positions every 15 ms. The mobile node receives these messages, associates the related RSSI value to each of them, and finally forwards them to the PC (4). The latter calculates a new estimate of the position only in the case the number of data messages is sufficient for the triangulation algorithm to work and periodically updates the tracking output sequence.

**Figure 12.**
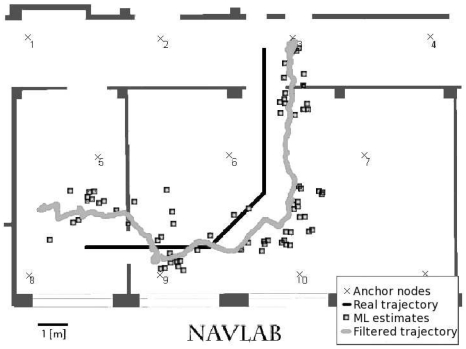
Tracking experiment. The reconstructed path is represented with a light grey solid line, non-filtered measurements are indicated with square markers; the actual path is shown with a solid black line. The sensor nodes of the fixed network are represented with crosses.

**Figure 13.**
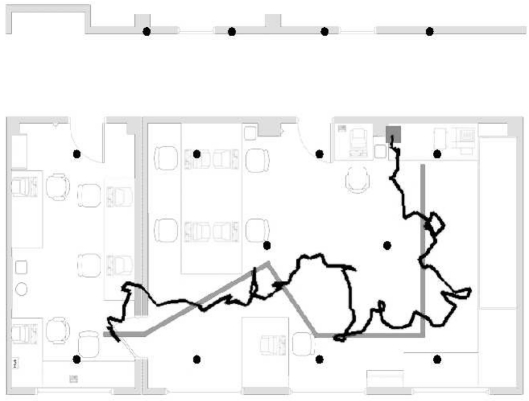
Tracking experiment. The reconstructed path is represented with a black solid line; the actual path is shown with a solid light grey line. The sensor nodes of the fixed network are represented with black dots.

In Figures 12-13 two results obtained from the localization algorithms are shown. These plots highlight the good accuracy in reproducing the correct path, and the role of the filtering procedures, that enforce spatial consistency of successive estimates, through the recursive temporal features of the EKF.

## 5. Conclusions

This paper has described some of the main activities that are being carried out within the context of the Italian WISE-WAI project [25], funded by the Cassa di Risparmio di Padova e Rovigo Foundation, Italy. The project, which aims at demonstrating the feasibility of large-size and wide-area wireless sensor networks, is now at an advanced level of progress: the working team has overseen the installation of part of the testbed in the areas of the Department of Information Engineering of the University of Padova, Italy, and has deployed fundamental services that will be used to control the network (e.g., wireless reprogramming), to adapt it to the specific environment it operates in (e.g., channel identification), and applications which require a high level of in-network interactions (such as localization and tracking). The design of both the protocols and the applications has been driven by an energy-efficiency paradigm, which led, e.g., to the use of efficient fountain codes for error recovery in wireless reprogramming, and to the use of a limited portion of the network for localization purposes.

The project is scheduled to end in June 2010. By that time, the network will cover a large area in the department buildings and will feature context-aware environmental monitoring and control functions, as well as more advanced applications based on localization, such as assisted navigation of buildings, which are currently being deployed.

## Figures and Tables

**Figure 2.**
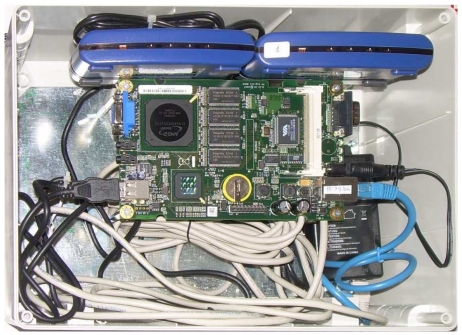
An example of Node Cluster Gateway (NCG), showing the embedded computer in the center, and the two USB 2.0-compliant hubs at the top.

**Figure 3.**
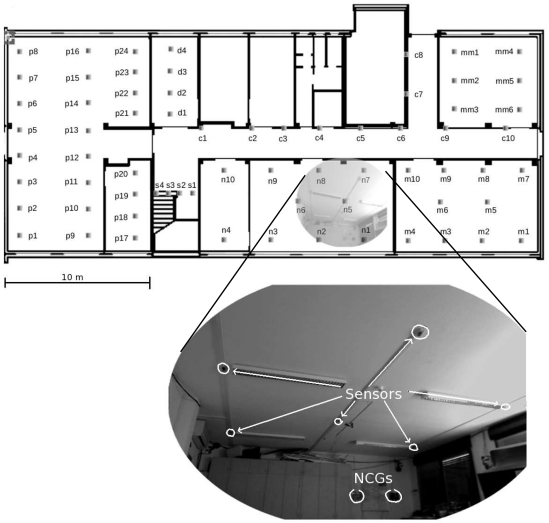
Node displacement map, with a close-up on the arrangement of nodes within one of the rooms.

**Figure 9.**
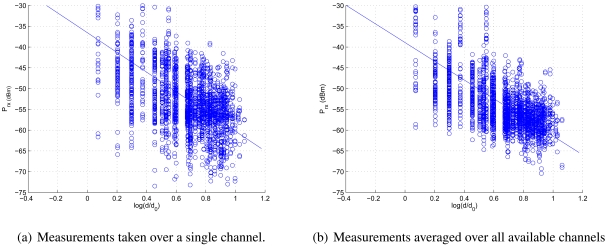
RSSI measurement campaigns for channel parameter identification.

**Table 1.** Channel parameters estimated for every considered ZigBee channel and for all channels.

Freq	2405	2410	2420	2435	2440	2445	2450	2455	2460	2465	2470	2475	2480	**ALL**

*K*	-27.2	-26.4	-25.9	-24.2	-22.8	-22.9	-22.3	-23.0	-22.4	-21.8	-21.0	-20.6	-20.0	**-23.2**
*η*	2.14	2.18	2.15	2.21	2.31	2.27	2.30	2.22	2.24	2.27	2.33	2.34	2.37	**2.25**
$\sigma_{\Psi_{i}}^{2}$	52.3	52.0	56.6	54.5	53.7	52.6	52.7	52.5	53.4	54.7	58.4	55.6	54.4	**35.4**
